# Biochemical Characterization and Function of Eight Microbial Type Terpene Synthases from Lycophyte *Selaginella moellendorffii*

**DOI:** 10.3390/ijms22020605

**Published:** 2021-01-09

**Authors:** Yapei Zhao, Tian Hu, Ruiqi Liu, Zhiqiang Hao, Guoyan Liang, Guanglin Li

**Affiliations:** Key Laboratory of Ministry of Education for Medicinal Plant Resource and Natural Pharmaceutical Chemistry, College of Life Sciences, Shaanxi Normal University, Xi’an 710119, Shaanxi, China; yapeizhao@snnu.edu.cn (Y.Z.); hutian@snnu.edu.cn (T.H.); ruiqiliu@snnu.edu.cn (R.L.); haozhiqiang@snnu.edu.cn (Z.H.); lgy666@snnu.edu.cn (G.L.)

**Keywords:** lycophyte, SmMTPSLs, stress physiology, GC-MS, antibacterial

## Abstract

*Selaginella moellendorffii* is a lycophyte, a member of an ancient vascular plant lineage. Two distinct types of terpene synthase (*TPS*) genes were identified from this species, including *S. moellendorffii TPS* genes (*SmTPSs*) and *S. moellendorffii* microbial *TPS-*like genes (*SmMTPSLs*). The goal of this study was to investigate the biochemical functions of SmMTPSLs. Here, eight full-length *SmMTPSL* genes (*SmMTPSL5, -15, -19, -23, -33, -37, -46,* and *-47)* were functionally characterized from *S. moellendorffii*. *Escherichia coli-*expressed recombinant SmMTPSLs were tested for monoterpenes synthase and sesquiterpenes synthase activities. These enzymatic products were typical monoterpenes and sesquiterpenes that have been previous shown to be generated by typical plant TPSs when provided with geranyl diphosphate (GPP) and farnesyl diphosphate (FPP) as the substrates. Meanwhile, *SmMTPSL23*, *-33*, and *-37* were up-regulated when induced by alamethicin (ALA) and methyl jasmonate (MeJA), suggesting a role for these genes in plants response to abiotic stresses. Furthermore, this study pointed out that the terpenoids products of SmMTPSL23, -33, and -37 have an antibacterial effect on *Pseudomonas syringae* pv. *tomato* DC3000 and *Staphylococcus aureus*. Taken together, these results provide more information about the catalytic and biochemical function of SmMTPSLs in *S. moellendorffii* plants.

## 1. Introduction

Terpenoids are the largest class of specialized secondary metabolites, which are widely distributed in nature with large quantities and diverse structures [1]. Terpenoids form an essential part of direct and indirect defense systems against herbivores and pathogens [2]. They are the derivatives of isopentenyl diphosphate (IPP) and its allylic isomer dimethylallyl diphosphate (DMAPP), which are mainly produced by the cytosolic mevalonate (MVA) and the plastidial 2-C-methyl-D-erythritol-4-phosphate (MEP) pathways *in plants* [3,4,5]. The sequential head-to-tail condensation of IPP and DMAPP in the presence of prenyltransferases produces the immediate precursors of terpenes, geranyl diphosphate (GPP), farnesyl diphosphate (FPP), and geranylgeranyl diphosphate (GGPP), which are converted into monoterpenes (C_10_), sesquiterpenes (C_15_), and diterpenes (C_20_), respectively, by the activity of terpene synthases (TPSs) [6,7,8]. The structural analyses of TPSs show that a highly conserved aspartate-rich ‘DDxxD’ motif as well as another conserved ‘NSD/DTE’ motif is critical for binding and positioning the metal ion-substrate complex in the active site cavity [9].

In addition to the typical plant TPSs, a new type of microbial terpene synthase-like proteins (MTPSLs) has been found in previous studies, which occur widely in nonseed land plants [10]. Compared with typical plant TPSs that consist of either three domains (αβγ) or two domains (αβ) [9,11], MTPSLs contain only an α-domain. The MTPSL genes were initially identified in the genome of the lycophyte *Selaginella moellendorffii*, from whose 48 *MTPSL* genes were identified [12]. It was found that the genome of *S. moellendorfii* existed two kinds of *TPS* genes. The first kind was designated as *S. moellendorffii TPS* genes (*SmTPSs*), consisting of 18 members. The second kind was designated as *S. moellendorffii* microbial *TPS-*like genes (*SmMTPSLs*), consisting of 48 members [12]. Phylogenetic analysis indicated that SmTPSs were very closely related to typical seed plant TPSs while SmMTPSLs were more similar to microbial TPSs, in particular fungal TPSs [12]. Meanwhile, it had been proved that several representative SmTPSs encoded diterpene synthases while representative SmMTPSLs encoded monoterpene and sesquiterpene synthases [12]. In addition to the six *MTPSL* genes biochemically characterized from *S. moellendorffii* [12], *MTPSLs* have been successively characterized in other species, including liverworts [13], ferns [14], red algae [15], hornworts [16], and mosses [10].

Despite increasing knowledge of the anatomical and biochemical processes of induced terpenoid defenses in *S. moellendorffii*, detailed molecular and biochemical dissection of these complex defense systems is currently limited by lack of identification and functional characterization of the *SmMTPSL* genes involved. Six *SmMTPSL* genes were characterized in a previous study [12]. However, most *SmMTPSL* genes, their particular biochemical functions and their contribution to chemical defense remain to be discovered and functionally characterized. In the present study, we sequentially characterized another eight *SmMTPSL* genes to investigate their biological functions in nonseed plants. First, these eight full-length *SmMTPSL* genes were identified by analyzing the available genomic and transcriptomic resources from *S. moellendorffii.* Next, we analyzed the phylogenetic relatedness of eight *SmMTPSL* genes with other known *MTPSLs.* Furthermore, in vitro biochemical characterization were performed, suggesting that SmMTPSLs display monoterpene synthase and sesquiterpene synthase activities. In addition, expression analysis of three *SmMTPSL* genes showed that SmMTPSLs in *S. moellendorffii* is responsible for the biosynthesis of stress-induced terpenoids. Lastly, it had been demonstrated that the enzymatic products of SmMTPSLs have an anti-bacterial activity in vitro.

## 2. Results

### 2.1. Identification of SmMTPSLs in the S. moellendorffii Genome

In the previous search of the draft genome sequence of *S. moellendorffii*, eight putative full-length *SmMTPSL* genes were identified, which were named as *SmMTPSL5, -15, -19, -23, -33, -37, -46,* and *-47* [12]. In this study, the eight full-length *SmMTPSL* genes were successfully extracted from *S. moellendorffii* plants. The eight *SmMTPSL* genes were annotated to contain principally zero or one intron. Their ORF sequences encoded 366, 348, 368, 356, 372, 372, 323, and 366 deduced amino acids, respectively. The calculated molecular weight of proteins encoded by these *SmMTPSL* genes ranged 37.46–42.58 kDa (Table S1). Proteins encoded by *SmMTPSL* genes were considerably smaller than typical plant TPSs mainly as a result of their lack of β and γ domains [17].

**Figure 1:**
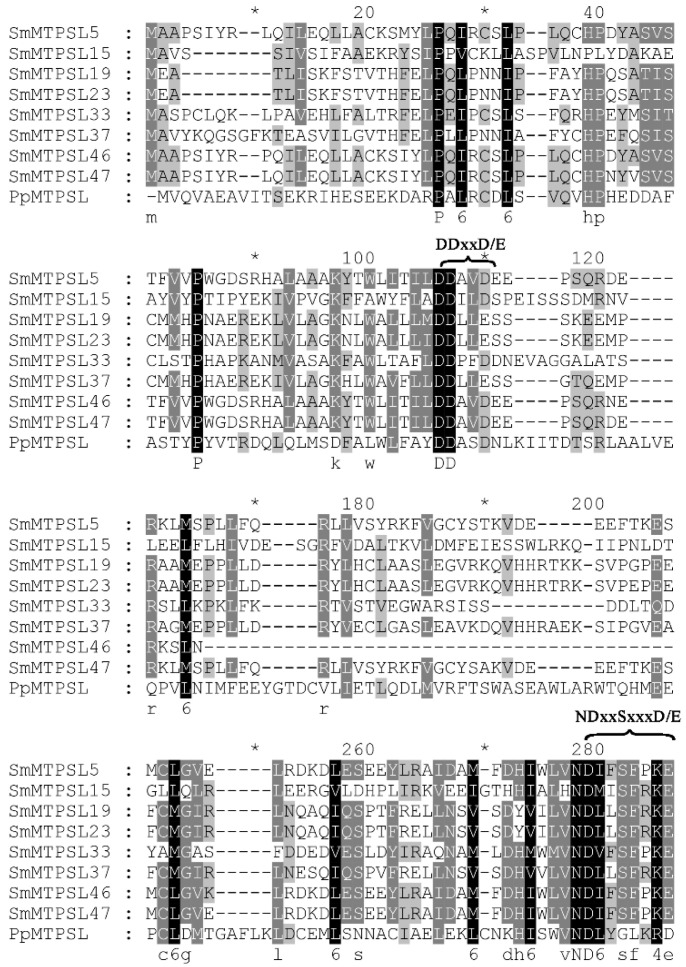
Amino acid sequence alignment of microbial terpene synthase-like proteins (MTPSLs). The Asp-rich ‘DDxxD’ motif and the ‘NSD/DTE’ motif, which were involved in metal-dependent ionization of the isoprenyl diphosphate substrate and conserved among all SmMTPSLs, are shown.

Multiple sequence alignment showed that the amino acid sequence of eight SmMTPSLs contained two features that are highly conserved among known TPSs. One was the Asp-rich ‘DDxxD’ motif, the other was the ‘NSD/DTE’ motif (Figure 1). The two conserved motifs were both involved in metal-dependent ionization of the isoprenyl diphosphate substrate [6]. The average identity and similarity of the eight *SmMTPSL* genes was 47% and 61%, respectively.

### 2.2. Phylogenetic Analysis of SmMTPSLs and Other Known TPSs

**Figure 2:**
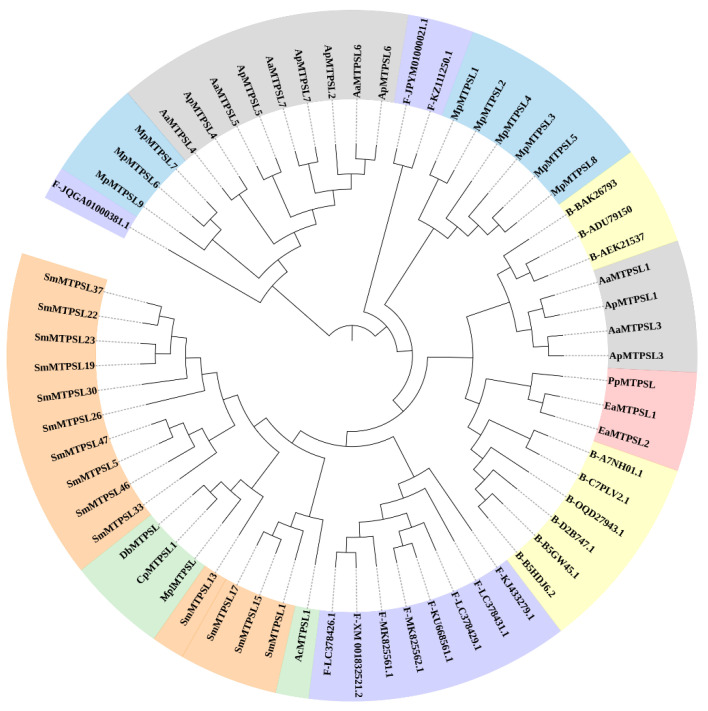
Phylogenetic tree of terpene synthase (TPSs) sequences. Sequences included are MTPSLs from *S. moellendorffii* (orange), ferns (green), red algae (pink), hornworts (gray), and liverworts (blue), and TPSs from fungi (purple) and bacteria (yellow). The tree was constructed with the maximum likelihood method and 1000 replications for bootstrapping. Bootstrap values are not drawn on any nodes with a value less than 70%. A list of genes GenBank accession numbers are given in Table S2.

To understand the evolutionary relatedness of SmMTPSLs with MTPSLs functionally characterized in nonseed plants and TPSs found in fungi and bacteria (Figure 2), the following TPSs were subjected to phylogenetic reconstruction, including 14 MTPSLs from *S. moellendorffii*, 13 MTPSLs from hornworts [16], 9 MTPSLs from liverworts [13], 4 MTPSLs from ferns [14], 3 MTPSLs from red algae [15], 11 TPSs from fungi, and 9 TPSs from bacteria [14]. Two major conclusions can be drawn. The first was that all SmMTPSLs clustered together with four ferns MTPSLs, which were DbTPSL, CpTPSL, MplTPSL, AcTPSL (Figure 2 and Figure S1), indicating that SmMTPSLs and MTPSLs from ferns might have a common ancestor. The second was that SmMTPSLs shared similarity with TPSs found in fungi and bacteria (Figure 2).

### 2.3. Structure Models and Molecular Docking of SmMTPSLs

**Figure 3:**
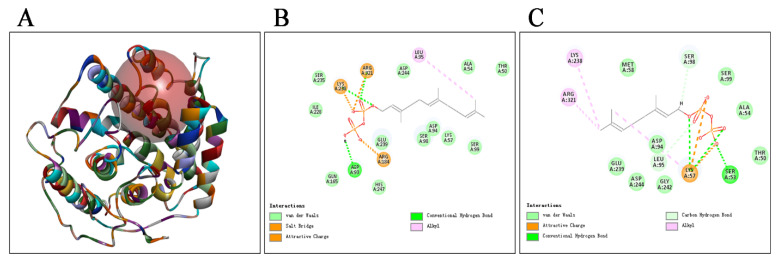
Structure model and molecular docking analysis of SmMTPSL19. (**A**) Three-dimensional model of SmMTPSL19. (**B**) Two-dimensional plan of SmMTPSL19 docking with farnesyl diphosphate (FPP). (**C**) Two-dimensional plan of SmMTPSL19 docking with geranyl diphosphate (GPP). The red circle represents the position of active pocket of receptor-ligand binding. The green dashed arrow represents the hydrogen bond between the atom on the main chain of the amino acid residue and the ligand. The pink dashed arrow represents the π-Alkyl interaction between the atom on the side chain of the amino acid residue and the ligand. The orange dashed line represents the π-Anion interaction force. The light green ellipse represents that amino acids and receptors form the van der Waals force.

To understand the structural basis underlying the functional divergence of SmMTPSLs, the structure models of eight SmMTPSL proteins were constructed using the I-TASSER Server. The Discovery Studio (DS) software was used to evaluate and score to select the best model, which was used for molecular docking, subsequently (Figure 3A). To predict the most stable pattern and active pocket of receptor-ligand binding, we performed SmMTPSLs molecular docking analysis with FPP and GPP by the DS software (Figure 3 and Figures S2–S8). The active pocket of receptor-ligand binding was highlighted in the red circle (Figure 3A). With semi-flexible docking, different conformations of ligand binding to the receptor produced a variety of docking results, and the group with the highest CDOCK score was selected as the best combination [18].

Taking SmMTPSL19 as an example, 17 amino acids had a mutual force with FPP according to the two-dimensional plan of docking (Figure 3B). Among them, amino acids K238, R321, and D93 with substrate FPP formed stable hydrogen bonds, which could help FPP find the entrance to the active site cavity and make the binding of ligand and receptor more stable. The Lys at 238 site and Arg at 321 site formed hydrogen bonds with oxygen atoms of FPP phosphate group, the Asp at 93 site formed hydrogen bonds with hydrogen atoms of FPP phosphate group. Besides, amino acids L95 formed the π-Alkyl interaction force with substrate FPP. Amino acids K238, R321, and R184 formed the π-Anion interaction force with substrate FPP. The remaining 12 amino acids interacted with FPP at the end of the active site cavity by van der Waals force. In addition, 14 amino acids had a mutual force with GPP (Figure 3C). Amino acids K57, S53, L95, and S98 with substrate GPP formed stable hydrogen bonds. The Lys at 57 site, Ser at 53 site, and Leu at 95 formed hydrogen bonds with oxygen atoms of GPP phosphate group, the Ser at 98 site formed hydrogen bonds with hydrogen atoms of GPP phosphate group. Besides, amino acids K57, K238, and R321 formed the π-Alkyl interaction force with substrate GPP. Amino acids K57 formed the π-Anion interaction force with substrate GPP. The remaining eight amino acids interacted with GPP at the end of the active site cavity by van der Waals force.

In this study, we also chose FPP and GPP as ligands to dock with the remaining seven SmMTPSLs (-5, -15, -23, -33, -37, -46, and -47), respectively. The docking generated the corresponding two-dimensional plan (Figures S2–S8). The docking results showed that each SmMTPSL synthase could successfully connect with the FPP or GPP, which was consistent with the facts that all eight SmMTPSLs can produce terpenoids using FPP or GPP as the substrates.

### 2.4. Catalytic Functions of SmMTPSLs

**Figure 4:**
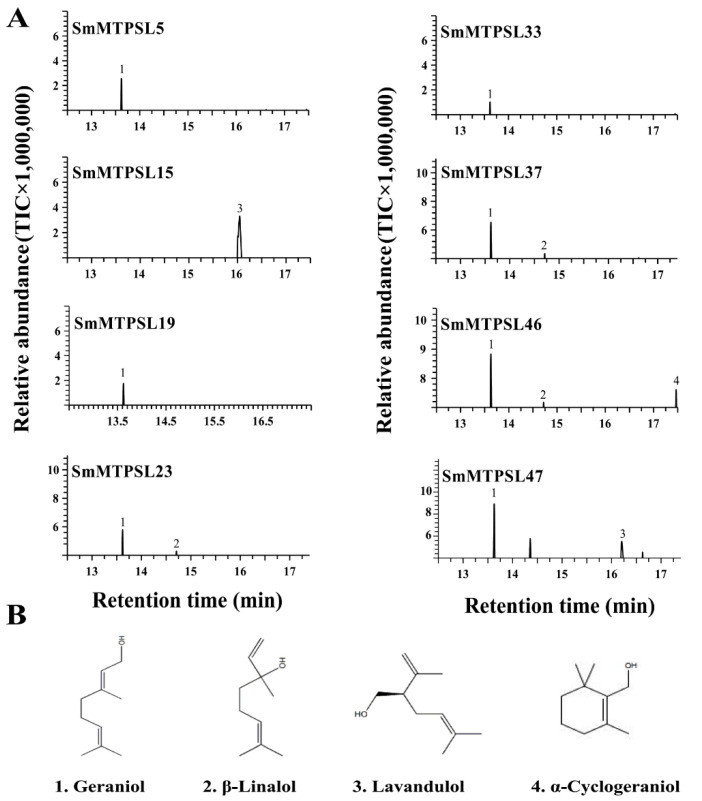
GC-MS of the products generated in vitro by eight recombinant SmMTPSLs proteins provided with GPP as the substrate. (**A**) Total ion chromatogram of the monoterpene products and (**B**) their structure. Products were identified as 1: geraniol; 2: β-linalool; 3: lavandulol; 4: a-cyclogeranio.

To investigate the catalytic activity of SmMTPSLs, all cloned *SmMTPSL* genes were heterologously expressed in *E. coli*. Recombinant SmMTPSLs proteins were tested with the potential substrates GPP or FPP. The eight SmMTPSs were all able to accept GPP as the substrate to produce monoterpenes (Figure 4A). SmMTPSL5, SmMTPSL19, and SmMTPSL33 catalyzed the formation of geraniol as a single product, while SmMTPSL15 yielded lavandulol as a major product. In contrast, the other three SmMTPSLs each produced multiple monoterpenes. SmMTPSL23 and SmMTPSL37 converted GPP to β-linalool and geraniol, when using GPP as the substrate. SmMTPSL46 produced three monoterpenes, including geraniol, β-linalool, and α-cyclogeraniol; whereas SmMTPSL47 could convert GPP into geraniol and lavandulol (Figure 4A).

**Figure 5:**
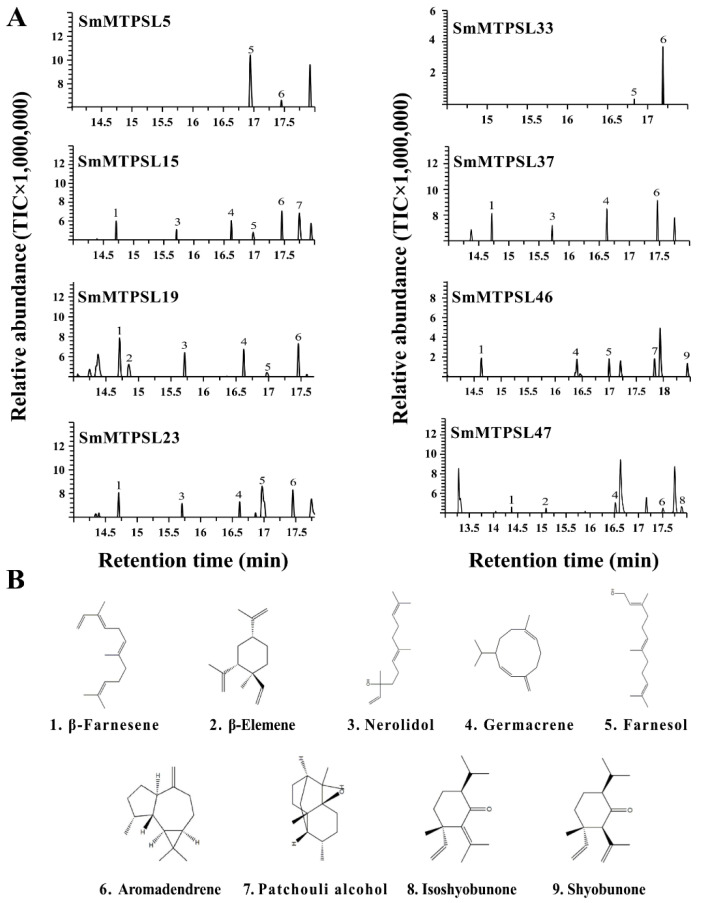
GC-MS of the products generated in vitro by eight recombinant SmMTPSLs proteins provided with FPP as the substrate. (**A**) Total ion chromatogram of the sesquiterpene products and (**B**) their structure. Products were identified as 1: β-farnesene; 2: β-elemene; 3: nerolidol; 4: germacrene; 5: farnesol; 6: aromadendrene; 7: patchouli alcohol; 8: isoshyobunone; 9: shyobunone.

Additionally, eight SmMTPSs all showed sesquiterpenes synthase activities with FPP as the substrate in vitro (Figure 5A). SmMTPSL5 produced two sesquiterpenes, including farnesol and aromadendrene, with the two products also produced by SmMTPSL33. Incubation of SmMTPSL15 with FPP catalyzed the formation of six sesquiterpenes predominantly aromadendrene along with β-farnesene, nerolidol, germacrene, farnesol, and patchouli alcohol. Upon incubation with FPP, SmMTPSL19 led to the production of six sesquiterpenes, including β-farnesene as a major product along with β-elemene, nerolidol, germacrene, farnesol, and aromadendrene while the SmMTPSL23 reactions with FPP resulted in the productions of five sesquiterpenes viz., β-farnesene, nerolidol, germacrene, farnesol, and aromadendrene, with the four of them also produced by SmMTPSL37. When SmMTPSL46 incubated with FPP, five sesquiterpene products were detected, viz., β-farnesene, germacrene, farnesol, patchouli alcohol, and shyobunone. When SmMTPSL47 incubated with FPP, five sesquiterpene products also were detected, including germacrene as a major product along with β-farnesene, β-elemene, aromadendrene, and isoshyobunone (Figure 5A).

### 2.5. Expression Analysis of SmMTPSL Genes in Response to Stress

**Figure 6:**
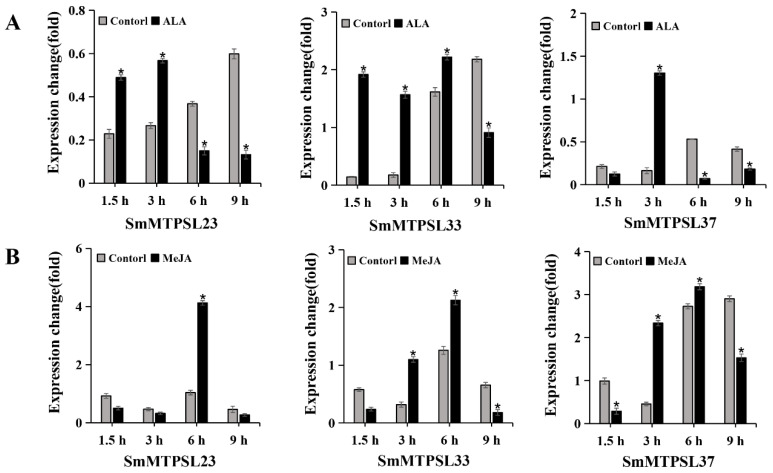
Gene expression of three *SmMTPSLs* in the leaves of *S. moellendorffii* treated by ALA (**A**) and MeJA (**B**) treatments. The expression level of *S. moellendorffii GAPDH* gene was used to normalize the expression of *SmMTSLs.* * indicates significance at the statistical level (*p* < 0.05) compared to the control.

*TPS* genes in land plants have diverse biological functions, particularly in defense against biotic and abiotic stresses [2]. To understand whether *SmMTPSL* genes have similar functions, relative gene expression analyses were conducted on *SmMTPSL23*, *-33*, and *-37* after alamethicin (ALA) or methyl jasmonate (MeJA), two signaling molecules in plant defense. The transcriptional response of the three *SmMTPSL* genes were analyzed using qRT-PCR. The three *SmMTPSL* genes were remarkably induced by the treatments. When plants were treated with ALA, *SmMTPSL23* showed the enhanced abundance at 1.5 and 3 h while *SmMTPSL33* showed the enhanced abundance at 1.5, 3, and 6 h compared to the control plants. *SmMTPSL37*, on the other hand, had its highest transcript abundance at 3 h. The expression level of *SmMTPSL23* in treated plants at 1.5 and 3 h were all 2-fold higher than the level in control plants. *SmMTPSL33* respectively showed 13.5-fold and 8.9-fold increased expression at 1.5 and 3 h while *SmMTPSL37* showed 7.9-fold increased expression at 3 h as compared to control plants (Figure 6A). Furthermore, when plants were treated with MeJA, *SmMTPSL23* had its highest transcript abundance at 6 h. *SmMTPSL33* and *SmMTPSL37*, on the other hand, showed the enhanced abundance at 3 and 6 h compared to the control plants. The expression level of *SmMTPSL23* was increased by approximately 4-fold at 6 h than the level in control plants. *SmMTPSL33* respectively showed 3.5-fold and 1.7-fold increased expression at 3 and 6 h while *SmMTPSL37* respectively showed 5.1-fold and 1.2-fold increased expression at 3 and 6 h as compared to control plants (Figure 6B).

### 2.6. Terpenoid Products from SmMTPSLs Have an Anti-Bacterial Activity In Vitro

**Figure 7:**
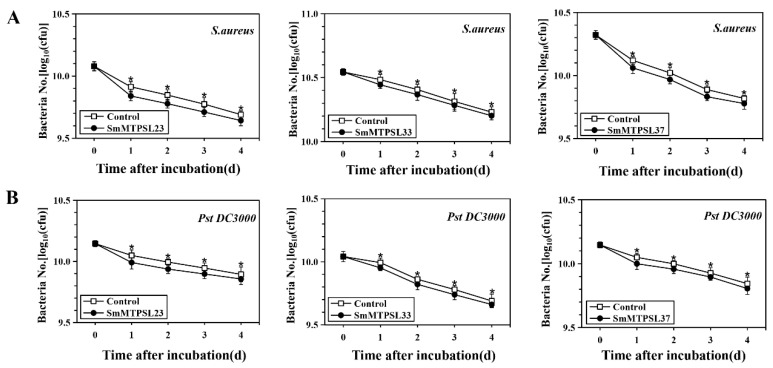
Inhibition effect of products from three SmMTPSLs on the growth of two pathogenic bacterial species: *S. aureus* (**A**) and *Pst* DC3000 (**B**). Three replicates of treatment and the control for each bacterial species was conducted. Data are presented as means ± standard deviations based on three replicates. * indicates significance at the statistical level (*p* < 0.05) compared to the control.

Terpenoids play an important role in the part of direct and indirect defense systems against herbivores and pathogens. Therefore, this work investigated the antibacterial properties and mode of action of terpenoid products, synthesized by three SmMTPSLs (SmMTPSL23, -33, and -37), against two pathogenic bacteria including *Pseudomonas syringae* pv. *tomato* (*Pst*) DC3000 and *Staphylococcus aureus*. Many terpenoid compounds identified to be constitutively emitted from plant leaves have been reported to have an anti-bacterial or anti-fungal effect on various species [19,20,21,22]. For example, ethanolic and hexane extracts from the aerial parts of *Juniperus lucayana*, with sesquiterpenes as the dominant compounds, were documented to have an anti-fungal effect on *Botrytis cinerea* [23]. In our study, it had been shown that enzymatic products of three SmMTPSLs (SmMTPSL23, -33, and -37) significantly inhibited the growth of *S. aureus*, with an inhibition rate of 13.6%, 7.6%, and 11.4%, respectively (Figure 7A). In addition, strains of the gram-negative bacterial pathogen, *Pst* DC3000, were also be used to test the anti-bacteria effects of reaction products. Compared with *S. aureus*, the enzymatic products of three *SmMTPSLs* showed a little weaker inhibition to the *Pst* DC3000, with an inhibition rate of 10.9%, 8.3%, and 8.9%, respectively (Figure 7B). These results showed that the enzymatic products of SmMTPSLs exhibited significant antimicrobial activity against tested bacterial, especially against *S. aureus*, gram-positive bacteria.

## 3. Discussion

In an attempt to identify genes of direct and putative indirect terpenoid defenses in *S. moellendorffii* and to provide a better foundation for phylogenetic analysis of the MTPSLs in nonseed plants family, eight full-length *SmMTPSL* genes were selected and functionally characterized from *S. moellendorffii*. The eight SmMTPSLs all contain two highly conserved motifs: ‘DDxxD’ and ‘NSD/DTE’ (Figure 1). Most MTPSLs, like typical plant TPSs, harbor a canonical aspartate-rich motif: ‘DDxxD’. The aspartate-rich motif functions in binding a cluster of three Mg^2+^ ions, which in term binds to the isoprenyl diphosphate substrates [24]. The binding leads to conformational changes of the active site, initiating the ionization and cyclization reaction. To the best of our knowledge, TPS function through divalent metal ion dependent generation of enzyme bound carbocation intermediates [25,26,27]. The phylogenetic analysis showed that all SmMTPSLs clustered with four MTPSLs from ferns and were similar to TPSs found in fungi and bacteria, which were consistent with the conclusion in the previous literature [12,14].

According to the docking result of SmMTPSL19 with FPP or GPP, the amino acids D93, K238, R321, K57, S53, and S98 were easy to form hydrogen bond with the ligand, making the binding of ligand and receptor more stable (Figure 3). The remaining structure models and molecular docking also made clear that the other seven SmMTPSLs could successfully connect with the FPP or GPP. These models and molecular docking have painted a picture of some of the amino acids involved in the active sites of these SmMTPSLs and provided some level of confidence for modeling of SmMTPSLs, which will direct future experiments to analyze the involvement of these amino acids in catalysis. Through this, we hope to pinpoint amino acids involved in catalysis and provide a basis for future site directed mutagenesis of SmMTPSLs.

As shown in our study, the eight SmMTPSLs were all determined to be functional, respectively, based on in vitro assays of the heterologously expressed proteins. All of them could use GPP or FPP as the substrate to display monoterpene synthase and sesquiterpene synthase activities (Figure 4 and Figure 5). Like previously characterized MTPSLs from *S. moellendorffii* [12], we note that all functional SmMTPSLs were also multi-product enzymes possessing sesquiterpene synthase activity in the present study. With some of them producing two or three sesquiterpenes, SmMTPSL15 appeared capable of producing as many as six sesquiterpenes. In this context, it is noteworthy that many typical plant TPSs are multi-product enzymes [28,29,30]. Another interesting observation is that the product profiles of SmMTPSL5 and SmMTPSL33 share strong similarities and both produce farnesol and aromadendrene (Figure 5). However, SmMTPSL5 and SmMTPSL33 share only 38% identity at the protein sequence level. It is thus an interesting question whether such similarity in catalytic activity reflects common ancestry or convergent evolution.

Terpenoids play significant ecological roles in the interactions between plants and stress conditions. As the critical gene in terpenoid biosynthesis, the expression level of *TPS* genes in different plants under various stress conditions were widely reported [31,32,33,34]. Both MeJA and ALA treatments regulated *SmMTPSL* genes expression (Figure 6). The expression level of *SmMTPSL23*, *-33*, and *-37* were enhanced after the MeJA treatment with the most notable enhancement occurred to *SmMTPSL33* (13.5-fold) at 1.5 h, followed by *SmMTPSL37* (7.9-fold) at 3 h. Besides, the most remarkably up-regulation was found for *SmMTPSL23* (4-fold) at 6 h, followed by *SmMTPSL33* (5.1-fold) at 3 h after the ALA treatment (Figure 6). The induction of *SmMTPSLs* expression in *S. moellendorffii* by MeJA or ALA supports that *TPS* genes play a role in defense against abiotic stresses. In addition, some genes shared the same induction alteration among two different treatments, suggesting that some genes may respond to the abiotic stresses in similar ways.

The antimicrobial activity of a variety of terpenoids has been described earlier by several authors [19,20,21,22,35,36]. The inhibitory effects of some pure terpenes and terpenoids on some bacteria and fungi for humans as well as phytopathogenic bacteria and fungi have already been reported. Based on the bacterial-inhibition assay [37,38,39,40], the present study pointed out that the enzymatic products of three SmMTPSLs (SmMTPSL23, -33, and -37) at the proper concentration reduced the growth of bacteria, especially against *S. aureus*, gram-positive bacteria (Figure 7). This phenomenon made clear that terpenoids products act as a chemical defense against pathogenic infection and provide a chemical interpretation for the varied resistance or tolerance. It is worth remembering that the three SmMTPSLs are multi-product enzymes, so it will be significant to further evaluate the anti-bacterial effects of terpenoid compounds individually and in combinations. Moreover, other in vitro studies need to confirm the antimicrobial activity of such sesquiterpenes towards a larger number of strains, which may guide future efforts to characterize the anti-bacteria effect of terpenoid products in *S. moellendorffii.*

## 4. Materials and Methods

### 4.1. Plant Growth and Treatment

*S. moellendorffii* plants were grown in a growth chamber under controlled conditions of 22 °C with a 12 h photoperiod and a photosynthetic photon flux of 100 μM m^−2^ s^−1^. Plants were used for experiments at the height of 5 inches [32]. In order to maximize the quantitative and qualitative volatile emission and upregulate expression of *SmMTPSL* genes, plants were sprayed with 100 μM MeJA or 0.1 mg/L ALA (dissolved in 0.1% (*v/v*) ethanol). Treated leaves were sampled at 1.5, 3, 6, and 9 h after treatment for RNA isolation.

### 4.2. Sequence Search and Phylogenetic Analysis

Transcriptomes of lycophyte *S. moellendorffii* were downloaded from the OneKP (https://sites.google.com/a/ualberta.ca/onekp/) [41], and the longest open reading frame for each transcript was identified and translated into a peptide. SmMTPSLs were identified from the peptides using Pfam model PF03936, which correspond to the conserved domains localized at the C termini of known TPSs [12]. The isoelectric point of the proteins and their signal peptide were analyzed on the websites of ScienceGateway (http://www.sciencegateway.org/tools/proteinmw.htm) and TargetP 1.1 Server (http://www.cbs.dtu.dk/services/TargetP), respectively. The setting parameter of TargetP was Organism group: Plant; predict scope: Perform cleavage site predictions. Multiple sequence alignment was performed with Clustal Omega and the final result was generated using the software GeneDoc. The phylogenetic tree was reconstructed with MEGA6.0 using the maximum likelihood phylogenies with 1000 bootstrap repetitions. Bayesian inference was calculated with MrBayes3.2.4. Four Markov chains were run for 2 runs from random starting trees for 2 million generations, and trees were sampled every 100 generations. The first one-fourth generations were discarded as burn-in. A majority rule consensus tree of all remaining trees was calculated. Branches that received bootstrap support for maximum likelihood (ML) and Bayesian posterior probabilities (BPP) greater or equal than 70% (ML) and 0.9 (BPP), respectively, were considered as significantly supported. ITOL was used to visualize the phylogenetic tree.

### 4.3. Homology-Based Structural Modeling and Molecular Docking

Homology-based structural modeling of SmMTPSLs was accomplished using I-TASSER Server [42]. Then the best structure models obtained were docked with FPP or GPP, using the Discovery Studio (DS) software [43].

### 4.4. Cloning of SmMTPSLs Full-length cDNA and Expression in E. coli

The total RNA was extracted from the leaves of *S. moellendorffii* using Plant RNA Isolation Kit (BEI-BEI Biotech, Zhengzhou, China) and cDNA was synthesized by PCR using the HIScript II Q RT SuperMix (Vazyme Biotech Co., Ltd., Nanjing, China) according to the manufacturer’s instructions. Then PCR products were cloned into the protein expression vector pCold-TF and were fully sequenced. All primers used were listed in Table S3. The constructs and insert-free pCold-TF (negative control) were used to transform *E. coli* strain BL21 (DE3) [44].

*E. coli* BL21 (DE3) containing target plasmid was preincubated overnight at 37 °C in Luria-Bertani (LB) media supplemented with 50 μg/mL chloramphenicol and 50 μg/mL ampicillin. The culture was transferred into 200 mL LB media and grown at 37 °C with shaking (200 rpm) until an optical density at 600 nm (OD600) of 0.5–0.7 [14]. Protein expression was then induced with 1 mM isopropyl-β-D-thiogalactopyranoside (IPTG) and cells were grown with shaking (200 rpm) at 15 °C for 24 h. Then the cells were harvested with centrifugation (5000 rpm, 5 min), resuspended in PBS buffer, and homogenized by sonication on ice. The recombinant proteins were purified by a Ni-NTA Sefinose His-bind column according to the manufacturer’s protocol. Protein concentrations were detected using the Bradford reagent (Amresco, Shanghai, China) with bovine serum albumin (BSA) as standard [45]. SDS-PAGE analysis was used to detect the purity of recombinant protein.

### 4.5. Identification of Terpenoid Products by GC-MS

To determine the catalytic activity of SmMTPSLs, enzyme assays were carried out in a 2 mL GC glass vial, containing 100 µL purified recombinant proteins and 100 µL substrates buffer (10 mM MgCl_2_, 0.2 mM Na_2_WO_4_, 0.1 mM NaF, and 10 μM FPP or GPP). Assays with the pCold-TF vector proteins without insert were used as control. A solid-phase micro-extraction (SPME) fiber was placed in the headspace of the vial over the reaction mixture for 1 h at 37 °C. Then, the SPME fiber was subsequently inserted into the gas chromatograph injector. Volatile components were analyzed by a Shimadzu 17A gas chromatograph coupled to a quadrupole mass selective detector (QP5050A; Shi-madzu, Columbia, MD). The separation was performed on an RTX-1MS column (30 m × 0.25 mm i.d. × 0.25 µm thick, Restek, Shimadzu). Electronic impact (EI) mode was at 70 eV. The initial temperature of the column box was 50 °C (hold for 3 min) and increased to 100 °C at a rate of 10 °C/min. Then the temperature was increased to 160 °C at a rate of 3 °C/min and then to 230 °C at a rate of 10 °C/min, and finally to 260 °C at a rate of 5 °C/min (hold for 3 min). The flow rate of the carrier gas (helium) was 7.8 mL/min. Terpenoids products were identified by comparison of their retention times and mass spectra with those of authentic standards analyzed under the same conditions. Compounds identification was based on similarity to library matches (NIST.11, NIST.11s).

### 4.6. Real-Time qPCR Analyses

The leaves of MeJA(ALA)-treated and control *S. moellendorffii* plants were used to extract the total RNA. Then, 2µg RNA was reverse transcribed for analyses of gene expression. Quantitative reverse transcription PCR (qRT-PCR) was performed in a 20µL reaction volume using SYBR Green dye. The expression level of *S. moellendorffii glyceraldehyde-3-phosphate dehydrogenase* (*GAPDH*) gene was used to normalize the expression of *SmMTPSLs.* The PCR program was 95 °C (3 min), followed by 45 cycles at 95 °C (10 s) and 60 °C (30 s). Specific primer pairs were designed with Primer 6.0 software. All primers used are listed in Table S3. The relative expression level of *SmTPSLs* genes was calculated using the 2^−∆∆CT^ method. Three biological replicates were analyzed.

### 4.7. Antibacterial Effect of Terpenoid Products

Two pathogenic bacteria, *Pst* DC3000 and *S. aureus*, were used for the test of anti-bacterial effect of terpenoid products. A 5 mL preculture of bacteria (*Pst* DC3000 and *S. aureus*, respectively) from a single colony was grown overnight at 30 °C in Luria-Bertani (LB) media. Then 100 µL purified SmMTPSLs protein (50 µg/mL) and 100 µL substrates buffer (10 mM MgCl_2_, 0.2 mM Na_2_WO_4_, 0.1 mM NaF, and 10 μM FPP) were incubated for 1 h at 37 °C. After 1 h, the 200 µL enzymatic products and 100 µL bacterial suspension were cultured in a 24-well cell culture plate. Besides, the wells with each bacterial suspension were added only substrates buffer as a control group. The 24-well cell culture plates were cultured for four days at 37 °C, and the number of colonies was recorded daily through the dilution gradient plate method. The inhibition rate of SmMTPSLs products on these two strains was calculated according to the following formula: (control group average colony-forming units (cfu)—treatment group average cfu)/control group average cfu × 100%. There were three replicates of each treatment and the control for each bacterial strain.

## Data Availability

Data is contained within the article or Supplementary Material.

## Supplementary Materials

The following are available online at https://www.mdpi.com/1422-0067/22/2/605/s1, Table S1: Basic characterization of SmMTPSLs; Table S2: The GenBank accession numbers of 43 characterized MTPSLs from nonseed plants and 20 TPSs from fungi and bacteria; Table S3: Primers used in this study; Figure S1: Phylogenetic tree of TPSs sequences; Figures S2–S8: Structure model and molecular docking analysis of SmMTPSL5, -15, -23, -33, -37, -46, and -47.
