# Supplementary material for: Biochemical Characterization and Function of Eight Microbial Type Terpene Synthases from Lycophyte Selaginella moellendorffii
Source: Int J Mol Sci. 2021 Jan 9;22(2):605. doi: 10.3390/ijms22020605 (PMC7826640; doi:10.3390/ijms22020605)
Supplement: Supplementary file 1 [file ijms-22-00605-s001.pdf]

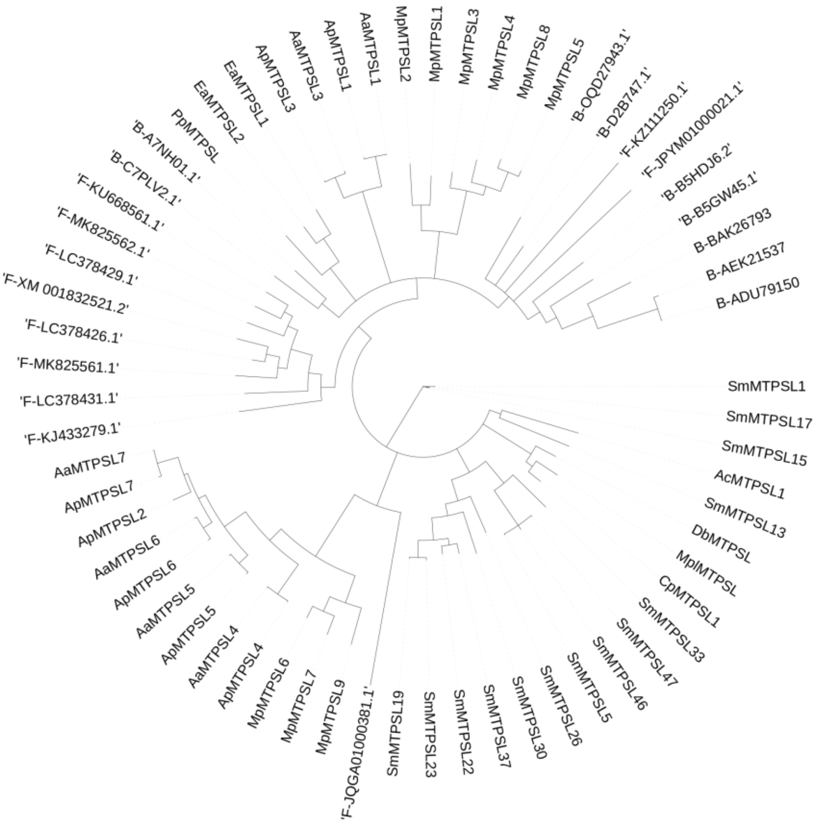

Figure S1. Phylogenetic tree of TPSs sequences. The tree was constructed with the bayesian method. A list of genes GenBank accession numbers were given in Table S2.

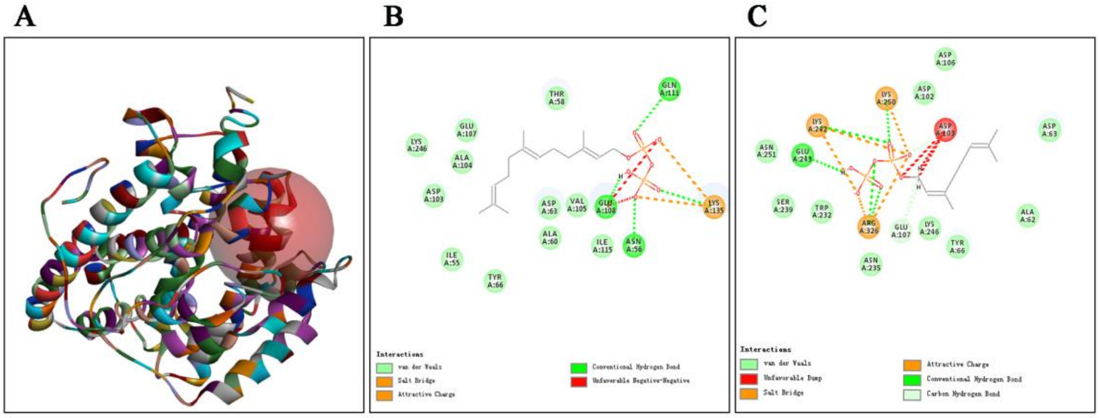

Figure S2. Structure model and molecular docking analysis of SmMTPSL5. (A) Three-dimensional model of SmMTPSL5. (B) Two-dimensional plan of SmMTPSL5 docking with FPP. (C) Two-dimensional plan of SmMTPSL5 docking with GPP. The red circle represents the the position of active pocket of receptor-ligand binding. The green dashed arrow represents the hydrogen bond between the atom on the main chain of the amino acid residue and the ligand. The pink dashed arrow represents the π-Alkyl interaction between the atom on the side chain of the amino acid residue and

the ligand. The orange dashed line represents the π-Anion interaction force. The light green ellipse represents that amino acids and receptors form the van der Waals force. The red dashed line represents the unfavorable negative force.

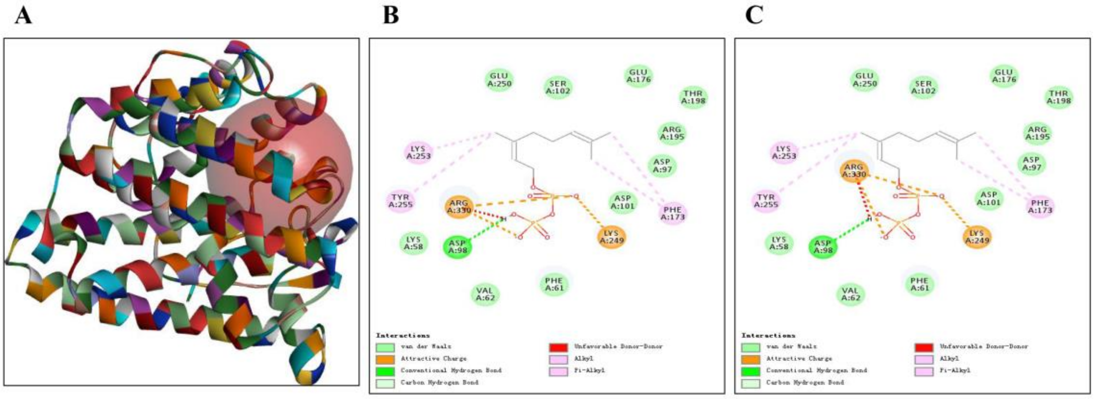

Figure S3. Structure model and molecular docking analysis of SmMTPSL15. (A) Three-dimensional model of SmMTPSL15. (B) Two-dimensional plan of SmMTPSL15 docking with FPP. (C) Two-dimensional plan of SmMTPSL15 docking with GPP. The red circle represents the the position of active pocket of receptor-ligand binding. The green dashed arrow represents the hydrogen bond between the atom on the main chain of the amino acid residue and the ligand. The pink dashed arrow represents the π-Alkyl interaction between the atom on the side chain of the amino acid residue and the ligand. The orange dashed line represents the π-Anion interaction force. The light green ellipse represents that amino acids and receptors form the van der Waals force. The red dashed line represents the unfavorable negative force.

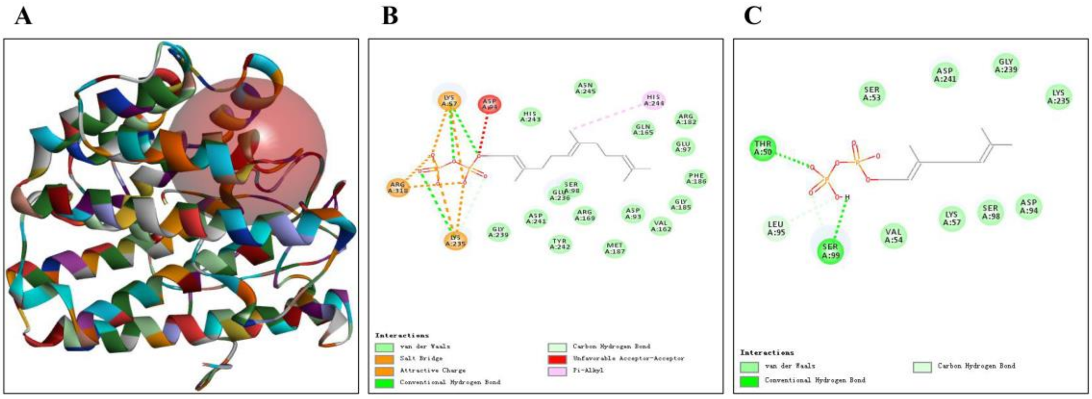

Figure S4. Structure model and molecular docking analysis of SmMTPSL23. (A) Three-dimensional model of SmMTPSL23. (B) Two-dimensional plan of SmMTPSL23 docking with FPP. (C) Two-dimensional plan of SmMTPSL23 docking

with GPP. The red circle represents the the position of active pocket of receptor-ligand binding. The green dashed arrow represents the hydrogen bond between the atom on the main chain of the amino acid residue and the ligand. The pink dashed arrow represents the π-Alkyl interaction between the atom on the side chain of the amino acid residue and the ligand. The orange dashed line represents the π-Anion interaction force. The light green ellipse represents that amino acids and receptors form the van der Waals force. The red dashed line represents the unfavorable negative force.

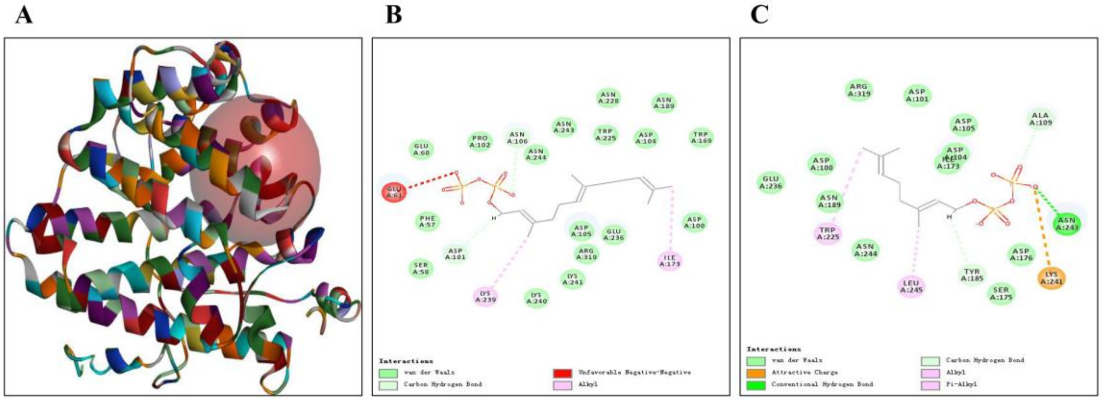

Figure S5. Structure model and molecular docking analysis of SmMTPSL33. (A) Three-dimensional model of SmMTPSL33. (B) Two-dimensional plan of SmMTPSL33 docking with FPP. (C) Two-dimensional plan of SmMTPSL33 docking with GPP. The red circle represents the the position of active pocket of receptor-ligand binding. The green dashed arrow represents the hydrogen bond between the atom on the main chain of the amino acid residue and the ligand. The pink dashed arrow represents the π-Alkyl interaction between the atom on the side chain of the amino acid residue and the ligand. The orange dashed line represents the π-Anion interaction force. The light green ellipse represents that amino acids and receptors form the van der Waals force. The red dashed line represents the unfavorable negative force.

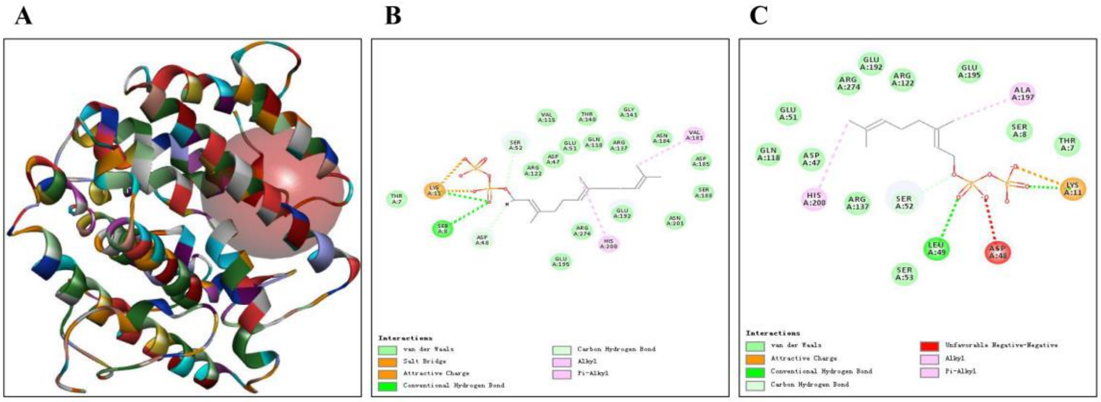

Figure S6. Structure model and molecular docking analysis of SmMTPSL37. (A) Three-dimensional model of SmMTPSL37. (B) Two-dimensional plan of SmMTPSL37 docking with FPP. (C) Two-dimensional plan of SmMTPSL37 docking with GPP. The red circle represents the the position of active pocket of receptor-ligand binding. The green dashed arrow represents the hydrogen bond between the atom on the main chain of the amino acid residue and the ligand. The pink dashed arrow represents the π-Alkyl interaction between the atom on the side chain of the amino acid residue and the ligand. The orange dashed line represents the π-Anion interaction force. The light green ellipse represents that amino acids and receptors form the van der Waals force. The red dashed line represents the unfavorable negative force.

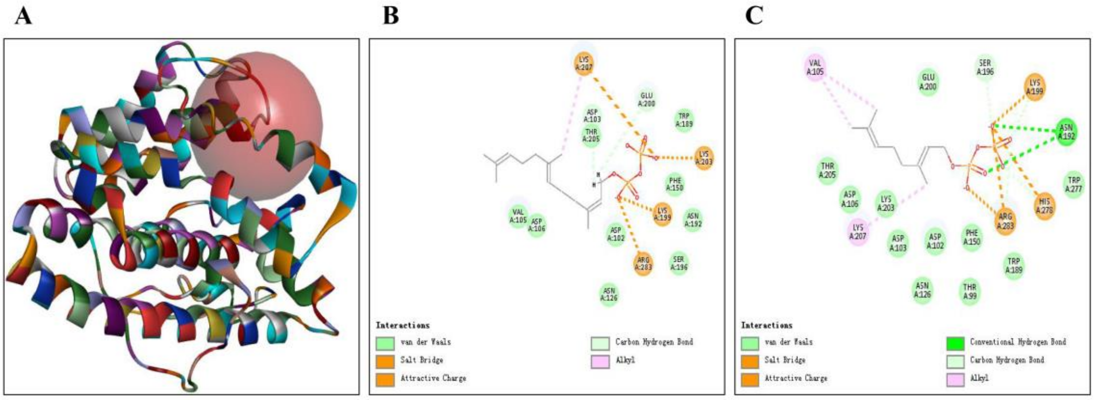

Figure S7. Structure model and molecular docking analysis of SmMTPSL46. (A) Three-dimensional model of SmMTPSL46. (B) Two-dimensional plan of SmMTPSL46 docking with FPP. (C) Two-dimensional plan of SmMTPSL46 docking with GPP. The red circle represents the the position of active pocket of receptor-ligand binding. The green dashed arrow represents the hydrogen bond between the atom on the main chain of the amino acid residue and the ligand. The pink dashed arrow

represents the π-Alkyl interaction between the atom on the side chain of the amino acid residue and the ligand. The orange dashed line represents the π-Anion interaction force. The light green ellipse represents that amino acids and receptors form the van der Waals force.

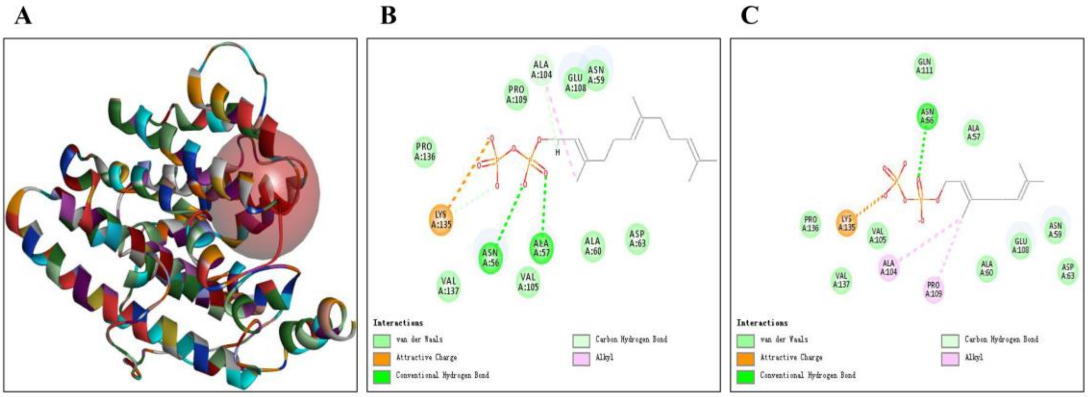

Figure S8. Structure model and molecular docking analysis of SmMTPSL47. (A) Three-dimensional model of SmMTPSL47. (B) Two-dimensional plan of SmMTPSL47 docking with FPP. (C) Two-dimensional plan of SmMTPSL47 docking with GPP. The red circle represents the the position of active pocket of receptor-ligand binding. The green dashed arrow represents the hydrogen bond between the atom on the main chain of the amino acid residue and the ligand. The pink dashed arrow represents the π-Alkyl interaction between the atom on the side chain of the amino acid residue and the ligand. The orange dashed line represents the π-Anion interaction force. The light green ellipse represents that amino acids and receptors form the van der Waals force.

Table S1 Basic characterization of SmMTPSLs.

Gene Name	Gene Length (bp)	Protein Length (Aa)	PI[a]	Loc[b]	Tple[c]	MW[d] (kDa)
SmMTPSL5	1101	366	6.47	S[e]	20	42.582
SmMTPSL15	1047	348	5.04	S	17	39.219
SmMTPSL19	1101	368	6.23	-[f]	-	41.221
SmMTPSL23	1072	356	6.51	-	-	40.344
SmMTPSL33	1119	372	6.02	S	31	42.472
SmMTPSL37	1119	372	5.53	S	33	41.921
SmMTPSL46	972	323	8.48	M[g]	113	37.460
SmMTPSL47	1101	366	6.64	S	20	42.467

[a] Isoelectric point; [b] Location of signal peptide; [c] Signal peptide; [d] Molecular weight; [e] Secretory pathway ; [f] Any other location; [g] Mitochondrion.

Table S2 The GenBank accession numbers of 43 characterized MTPSLs from nonseed plants and 20 TPSs from fungi and bacteria.

Gene name	Accession No. in GenBank	Species
SmMTPSL1	XP_002960898.1	*Selaginella moellendorffii*
SmMTPSL5	XP_002964134.1	*Selaginella moellendorffii*
SmMTPSL13	XP_002971857.1	*Selaginella moellendorffii*
SmMTPSL15	XP_002971976.1	*Selaginella moellendorffii*
SmMTPSL17	XP_024532655.1	*Selaginella moellendorffii*
SmMTPSL19	XP_002972941.1	*Selaginella moellendorffii*
SmMTPSL22	XP_002972952.1	*Selaginella moellendorffii*
SmMTPSL23	XP_002972730.2	*Selaginella moellendorffii*
SmMTPSL26	XP_024535054.1	*Selaginella moellendorffii*
SmMTPSL30	XP_002977567.1	*Selaginella moellendorffii*
SmMTPSL33	XP_024519169.1	*Selaginella moellendorffii*
SmMTPSL37	XP_024517709.1	*Selaginella moellendorffii*
SmMTPSL46	XP_002993173.1	*Selaginella moellendorffii*
SmMTPSL47	XP_002993175.1	*Selaginella moellendorffii*
AaMTPSL1	MF417641	*Anthoceros agrestis*
AaMTPSL3	MF417642	*Anthoceros agrestis*
AaMTPSL4	MF417643	*Anthoceros agrestis*
AaMTPSL5	MF417644	*Anthoceros agrestis*
AaMTPSL6	MF417645	*Anthoceros agrestis*
AaMTPSL7	MF417646	*Anthoceros agrestis*
ApMTPSL1	MF417637	*Anthoceros punctatus*
ApMTPSL2	MF417647	*Anthoceros punctatus*
ApMTPSL3	MF417638	*Anthoceros punctatus*
ApMTPSL4	MF417639	*Anthoceros punctatus*
ApMTPSL5	MF417640	*Anthoceros punctatus*
ApMTPSL6	MF417636	*Anthoceros punctatus*
ApMTPSL7	APB88778	*Anthoceros punctatus*
MpMTPSL1	APP91786	*Marchantia polymorpha*
MpMTPSL2	APP91787	*Marchantia polymorpha*
MpMTPSL3	APP91788	*Marchantia polymorpha*
MpMTPSL4	APP91789	*Marchantia polymorpha*
MpMTPSL5	APP91790	*Marchantia polymorpha*
MpMTPSL6	APP91791	*Marchantia polymorpha*
MpMTPSL7	APP91792	*Marchantia polymorpha*
MpMTPSL8	APP91793	*Marchantia polymorpha*
MpMTPSL9	APP91797	*Marchantia polymorpha*
PpMTPSL	MG262475	*Porphyridium purpureum*
EaMTPSL1	MH304416	*Erythrolobus australicus*
EaMTPSL2	MH304417	*Erythrolobus australicus*

Table S2 (continued)

Gene name	Accession No. in GenBank	Species
AcMTPSL1	MN868308	*Adiantum capillus-veneris*
CpMTPSL1	MN868309	*Cyclosorus parasiticu*
DbMTPSL	MN868310	*Drynaria bonii*
MplMTPSL	MN868311	*Microlepia platyphylla*
F-KU668561.1	KU668561.1	Fungi
F-XM_001832521.2	XM_001832521.2	Fungi
F-JPYM01000021.1	JPYM01000021.1	Fungi
F-JQGA01000381.1	JQGA01000381.1	Fungi
F-KJ433279.1	KJ433279.1	Fungi
F-KZ111250.1	KZ111250.1	Fungi
F-LC378426.1	LC378426.1	Fungi
F-LC378429.1	LC378429.1	Fungi
F-LC378431.1	LC378431.1	Fungi
F-MK825561.1	MK825561.1	Fungi
F-MK825562.1	MK825562.1	Fungi
B-A7NH01.1	A7NH01.1	bacteria
B-ADU79150	ADU79150	bacteria
B-AEK21537	AEK21537	bacteria
B-B5GW45.1	B5GW45.1	bacteria
B-B5HDJ6.2	B5HDJ6.2	bacteria
B-BAK26793	BAK26793	bacteria
B-C7PLV2.1	C7PLV2.1	bacteria
B-D2B747.1	D2B747.1	bacteria
B-OQD27943.1	OQD27943.1	bacteria

Table S3 Primers used in this study

Primer name	Primer sequences 5'-3'
**For gene cloning**	
SmMTPSL5-F	ATGGCCGCGCCTTCTATCTATC
SmMTPSL5-R	TCAGACTTGCAGATGGGGATGAGT
SmMTPSL15-F	ATGGCTGTTTCATCCATTGTGAGCAT
SmMTPSL15-R	CTAGAGTTGGAAGGTCGCTGATTTTTC
SmMTPSL19-F	ATGGAGGCCACTTTGATCTCCAAAT
SmMTPSL19-R	TCAAGGCAGTGGAACCGGAACT
SmMTPSL23-F	ATGGAGGCCACTTTGATCTCCAAAT
SmMTPSL23-R	GGGGCGAGCCTTGATGGAT
SmMTPSL33-F	ATGGCAAGTCCGTGTTTACAGAAGC
SmMTPSL33-R	TATCAAGTTTGATAAATTTTGGATCTGGTTGAG
SmMTPSL37-F	ATGAGAATCGCCGACGAGACTTC
SmMTPSL37-R	TATAGTTCCTGGTCTGGGTGATGC
SmMTPSL46-F	ATGGCCGCGCCTTCTATCTATCGT
SmMTPSL46-R	TCAGACTTGCAGATGGGGATGAGTTG
SmMTPSL47-F	ATGGCCGCGCCTTCTATCTATCGTC
SmMTPSL47-R	TCAAACTTGCAGATGGGGATGAGTTGGT
**For Real time PCR**	
SmMTPSL23-F	GGATGGAGCTATCCGGAATCCTGT
SmMTPSL23-R	GCAAGTAGCGGTCCAGCAAAGGT
SmMTPSL33-F	TCTTAGCCTCTGCTATGGAACCGC
SmMTPSL33-R	GGCTTCAACAGAGACCTCAAATCCTTC
SmMTPSL37-F	GAGTGGAAGCTTACAAGCTTTCCCG
SmMTPSL37-R	CTTTCGTTGAGGCGGATTCCCATAC
GAPDH-F	TCCATGGAAAGTGGAAGAGGGAGGATG
GAPDH-R	CCATGGGATATCTGCGGGATCCTTG
